# Permeability of a Zinc-Methacrylate-Based Self-Polishing Copolymer for Use in Antifouling Coating Materials by Molecular Dynamics Simulations

**DOI:** 10.3390/nano11113141

**Published:** 2021-11-21

**Authors:** Sung Hyun Kwon, Inwon Lee, Hyun Park, Seung Geol Lee

**Affiliations:** 1 School of Chemical Engineering, Pusan National University, Busan 46241, Korea; sunghyun.kwon@pusan.ac.kr; 2 Global Core Research Centre for Ships and Offshore Plants (GCRC-SOP), Pusan National University, Busan 46241, Korea; inwon@pusan.ac.kr; 3 Department of Naval Architecture and Ocean Engineering, Pusan National University, Busan 46241, Korea; 4 Department of Organic Material Science and Engineering, Pusan National University, Busan 46241, Korea

**Keywords:** molecular dynamics, self-polishing copolymer, antifouling agent, zinc methacrylate, H_2_O permeability

## Abstract

Molecular dynamics simulations were used to investigate the solubility and permeability of H_2_O in a self-polishing copolymer (SPC) with two zinc methacrylate (ZMA) contents (Z2: 2 mol% ZMA; Z16: 16 mol% ZMA) and ethyl acrylate, methyl methacrylate, 2-methoxyethyl acrylate, and butyl acrylate as antifouling agents. Water was found to be more soluble in hydrated Z16 than Z2 because ZMA interacts strongly with H_2_O. In contrast, the diffusion coefficient of H_2_O in Z16 is lower than that of Z2 because H_2_O molecules are more constrained in the former due to strong ZMA/H_2_O interactions. Z16 was found to be significantly more permeable than Z2 over time. The SPC hydrated region in Z2 tends to expand toward the SPC region, while the analogous region in Z16 swelled toward both the SPC and H_2_O regions to leach SPC owing to the higher permeation of H2O into the SPC. These results reveal that H_2_O permeability can be controlled by adjusting the ZMA content, which provides insight into antifouling performance.

## 1. Introduction

Marine biofouling occurs on the surfaces of marine platforms and ships in seawater because seawater contains various types of marine organisms, including seaweed, bacteria, microalgae, and barnacles [1,2]. In particular, the adsorption of marine organisms under the hulls of vessels, and platforms increases surface roughness [3,4]. Surface coating methods are mainly used to protect the surfaces of marine platforms and ships, thereby preventing the unwanted accumulation of marine organisms. Moreover, chemical, physical, and biological antifouling coating agents have been developed [2,5,6,7]. In particular, antifouling coating agents consist of self-polishing copolymers (SPCs) that are mainly composed of hydrolysable polymers because the pendant groups decompose by hydrolysis in seawater to remove unwanted marine organisms [2,4,8].

Tributyltin (TBT) was among the earliest antifouling agents developed and remains one of the most effective agents for use in SPCs that protect the surfaces of marine platforms and ships [1,2,9,10]. The pH and NaCl concentration of seawater affect the polishing and leaching behaviour of TBT in an SPC when applied as an antifouling coating agent [11]. However, TBT-based SPCs were banned in 2003 because TBT is toxic to the marine environment [1]. Therefore, TBT has been replaced by environmentally safe antifouling agents for use in SPCs. For example, acrylate esters are still used in SPCs because they act as antifouling agents, while hydrolysable pendant groups, such as TBT, have been substituted with environmentally safe components. In particular, zinc methacrylate (ZMA) [12,13,14], copper methacrylate (CMA) [2,15], and triisopropylsilyl acrylate (TIPSA) [8,14] have been investigated as hydrolysable pendant antifouling agents [1].

The polishing and leaching behaviour of an SPC is affected by the type and content of the SPC pendant group. Kim et al. [12] reported that the ZMA content in an SPC is important to its self-polishing behaviour, with increasing SPC leaching observed with increasing ZMA content. In particular, the leaching rate affects the erosion rate, as the SPC erosion rate is closely related to the leaching behaviour of the SPC. Therefore, comparing and analysing the decomposition and leaching characteristics of the hydrolysable pendant groups in an SPC are essential for achieving better SPC antifouling performance. In addition, while understanding how the ZMA content affects the polishing behaviour of the SPC is important, analysing the decomposition characteristics of the hydrolysable pendant groups in the SPC is essential when investigating the antifouling performance of an SPC.

For example, Kwon et al. [16] investigated the decomposition mechanism of an SPC with different pendant groups, including TBT, ZMA, CMA, and TIPSA, using density functional theory (DFT) and a computational simulation method. Their results revealed that ZMA and CMA afford lower activation energies than TBT and TIPSA and that the former pair can also be polished faster than the latter. However, while the type of pendant group affects the permeation characteristics of seawater into an SPC, various hydrophobic monomer compositions in the SPC are also important because Kiil and Yebra [17] reported that antifouling protection occurs with SPC leaching through the diffusion of seawater into the SPC polymer matrix. Therefore, various polymer matrices with different pendant-group contents and hydrophobic monomer compositions need to be investigated to fully reveal the decomposition and leaching behaviour of an SPC. Moreover, detailed motion at the molecular level needs to be investigated during seawater permeation into an SPC to understand its characteristics. However, few fully atomistic systematic studies have been reported.

In this context, molecular dynamics (MD) simulations can be used to calculate detailed molecular information, such as diffusion and permeation properties, for ZMA-based SPC systems. We chose ZMA-based SPCs because ZMA is a hydrolysable pendant group that has replaced TBT [12,14,18]; its erosion rate is also significantly affected by seawater conditions (pH and NaCl concentration) [11], as well as the ZMA concentration [12]. In particular, the ZMA content strongly affects self-polishing behaviour by altering the leaching and erosion rates. We expected MD simulations to be useful for investigating the diffusion behaviour and permeability of water-based media into an SPC, which significantly affects the polishing and leaching characteristics of the SPC. We determined the diffusion coefficient and permeability of water molecules at two ZMA contents and compared the permeability and leaching behaviour of the SPC because the ZMA content affects the erosion rate by preventing the unwanted accumulation of marine organisms [12]. For this purpose, two ZMA contents (2 and 16 mol%) were constructed with hydrophobic monomer compositions, such as ethyl acrylate (EA), methyl methacrylate (MMA), 2-methoxyethyl acrylate (2-MTA), and butyl acrylate (n-BMA) in the SPC. The density distributions of water molecules in the SPC were calculated to determine water solubility at the two ZMA contents. Moreover, water permeability was calculated using the diffusion coefficient and solubility of water molecules in the SPC. In addition, the morphologies of SPC in water were captured to investigate the swelling and leaching features of hydrated SPC as the ZMA content was changed at the molecular level.

## 2. Computational Details

### 2.1. Model Preparation

**Figure 1:**
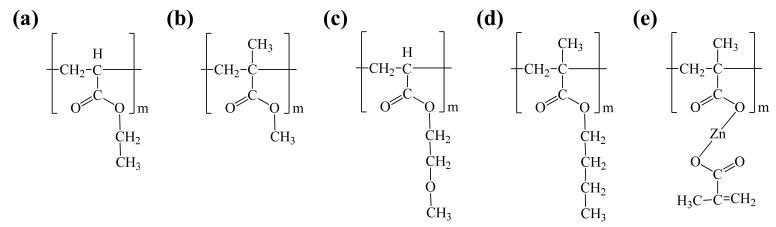
Chemical structures of the organic compounds in the SPC: (**a**) EA, (**b**) MMA, (**c**) 2-MTA, (**d**) n-BMA, and (**e**) ZMA.

The molecules in the SPC were simulated using full atomistic schemes with ethyl acrylate (EA), methyl methacrylate (MMA), 2-methoxyethyl acrylate (2-MTA), butyl acrylate (n-BMA), and ZMA because the SPC consists of hydrolysable pendant groups but also various hydrophobic monomers. In particular, several studies have shown that an SPC mainly consists of EA, MMA, 2-MTA, n-BMA, and ZMA [12,19,20], the structures of which are shown in Figure 1. SPC systems with varying ZMA contents were prepared using the model preparation step, with the molar proportions of EA, MMA, 2-MTA, n-BMA, and ZMA, as antifouling agents in the SPC listed in Table 1; these proportions were adopted based on experimental formulations [12]. The proportions of ZMA and MMA were altered to compare H_2_O permeabilities and SPC leaching behaviour. Two SPC models were prepared—one with low ZMA content (2 mol%; Z2) and the other with high ZMA content (16 mol%; Z16), with EA, MMA, 2-MTA, n-BMA, and ZMA evenly distributed in an SPC with 50 degrees of polymerisation. To charge the SPC, density functional theory (DFT) calculations were performed using the DMol^3^ modules in the Materials Studio software package [21] and Mulliken charge analysis [22]. The Perdew–Burke–Ernzerhof (GGA–PBE) functional, and a double numerical basis set with polarisation (DNP) functions were used in the DFT calculations along with a generalised gradient approximation [23]. F3C-water-model charges [24] were used in the MD simulations. Five atomic Fe(100) layers were used to construct an Fe slab 48.729 × 48.729 × 300.000 Å in size, with periodic boundary conditions (PBCs) applied in all directions.

### 2.2. MD Simulations

The large-scale atomic/molecular massively parallel simulator (LAMMPS) code, developed by Plimpton et al. [25] was used for full atomistic MD simulations. The modified DREDING force field [26] was used for SPC, and the F3C force field [24] was used for H_2_O molecules because these force fields have been successfully used to describe various organic materials, including polymers [27,28,29,30,31].

Total potential energies were calculated using Equation (1).

(1) $$E_{total}=E_{vdW}+E_{Q}+E_{bond}+E_{angle}+E_{torsion}+E_{inversion}$$

where $E_{total}$, $E_{vdW}$, $E_{Q}$, $E_{bond}$, $E_{angle}$, $E_{torsion}$, and $E_{inversion}$ are total system, van der Waals, electrostatic, bond-stretching, angle-bending, torsion, and inversion energies in the SPC system, respectively. The velocity–Verlet integration algorithm [32] with 1 fs time steps was used to integrate the equations of atomic motion in the MD simulations. Electrostatic interactions in the SPC systems were calculated using the particle–particle, particle–mesh (PPPM) method [33].

To calculate the density profiles and diffusion behaviour of H_2_O in the SPC, model bulk SPC states were constructed using six SPC chains and 0, 10, 20, and 30 wt% molecular H_2_O using Monte Carlo (MC) simulations [21]. It should be noted that we used H_2_O rather than all of the seawater components because modeling the complex seawater medium is very computationally expensive [34]. Annealing was performed within the MD simulations to obtain equilibrated structures of bulk SPC for density analysis; annealing is commonly used to accelerate the equilibration process in MD studies [35,36,37,38]. The annealing steps involve (a) gradually increasing the temperature of the initial SPC structure with H_2_O from 0 K to 298.15 K over 300 ps by canonical ensemble (NVT) simulation, (b) gradually increasing the temperature from 298.15 K to 1000 K while expanding the volume of the SPC structure to 200% of the initial volume, (c) simulating the SPC structure at 1000 K for 100 ps, (d) gradually returning the volume of the SPC structure while gradually decreasing the temperature from 1000 K to 298.15 K, (e) repeating steps (b) to (d) three times, and finally (f) performing an NVT simulation for 100 ps, followed by 500 ps of isothermal–isobaric ensemble (NPT) simulation at 1 atm. NPT simulations were performed at 298.15 K and 1 atm for 10 ns at the end of the annealing process. Data were collected through an additional 5 ns of NPT simulation following equilibration. More than five identical models were simulated in each case, with average data reported.

The Fe slab models used to investigate H_2_O permeation behaviour in the SPC were fabricated by randomly constructing 10 SPC chains by MC simulation [21], after which they were annealed to relax the molecular structure on Fe(100) surface by (a) gradually increasing the temperature of the SPC on the Fe(100) slab from 0 K to 298.15 K over 300 ps by NVT MD simulation, (b) linearly increasing the temperature from 298.15 K to 1000 K over 1 ns and maintaining the temperature at 1000 K for 1 ns of NVT simulation to obtain the relaxed SPC structure on the Fe(100) slab, (c) decreasing the temperature of the structure from 1000 K to 298.15 K over 1 ns, (d) repeating all of the abovementioned annealing steps three times to obtain fully relaxed molecular structures, (e) adding 2102 H_2_O molecules [39] to the top of the dry SPC state to investigate the relationships between H_2_O solubility in the SPC and the ZMA and MMA contents, and finally, (f) 150 ns NVT simulation at 298.15 K to obtain equilibrated structures, with data collected for H_2_O solubility calculations.

## 3. Results and Discussion

### 3.1. SPC Density Analysis

**Figure 2:**
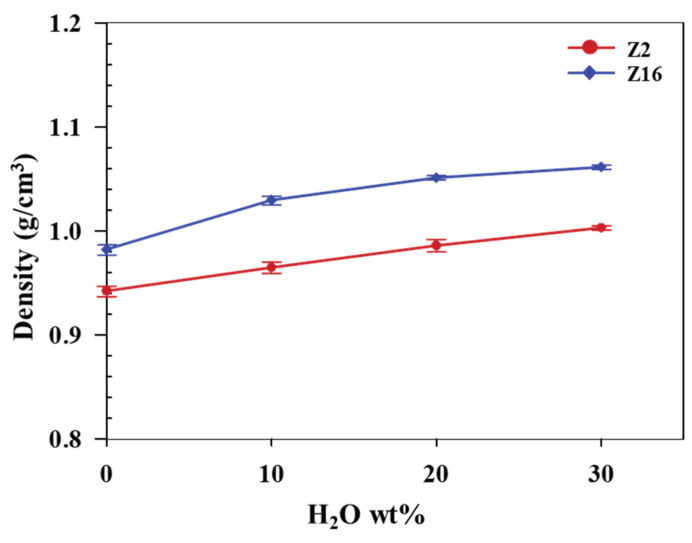
Z2 and Z16 densities as functions of H_2_O content.

Figure 2 shows the density profiles of Z2 and Z16 in their bulk SPC states with various H_2_O contents. The density of the dry Z2 state was determined to be 0.942 ± 0.005 g/cm^3^, while Z16 exhibited a value of 0.982 ± 0.005 g/cm^3^. On the other hand, the hydrated states of Z2 at 298.15 K exhibited densities of 0.965 ± 0.005 g/cm^3^ (10 wt% H_2_O), 0.986 ± 0.006 g/cm^3^ (20 wt% H_2_O), and 1.003 ± 0.004 g/cm^3^ (30 wt% H_2_O). The analogous values for the hydrated state of Z16 at 298.15 K are 1.029 ± 0.004 g/cm^3^ (10 wt% H_2_O), 1.051 ± 0.002 g/cm^3^ (20 wt% H_2_O), and 1.061 ± 0.002 g/cm^3^ (30 wt% H_2_O). The densities of Z2 and Z16 increase with increasing water content, which indicates that the H_2_O molecules are well located within the SPCs. The density of the dry and hydrated states of Z16 are higher than those of Z2 because ZMA (1.40 g/cm^3^ [40]) is denser than MMA (0.94 g/cm^3^) [41].

### 3.2. H_2_O Solubility in SPC

**Figure 3:**
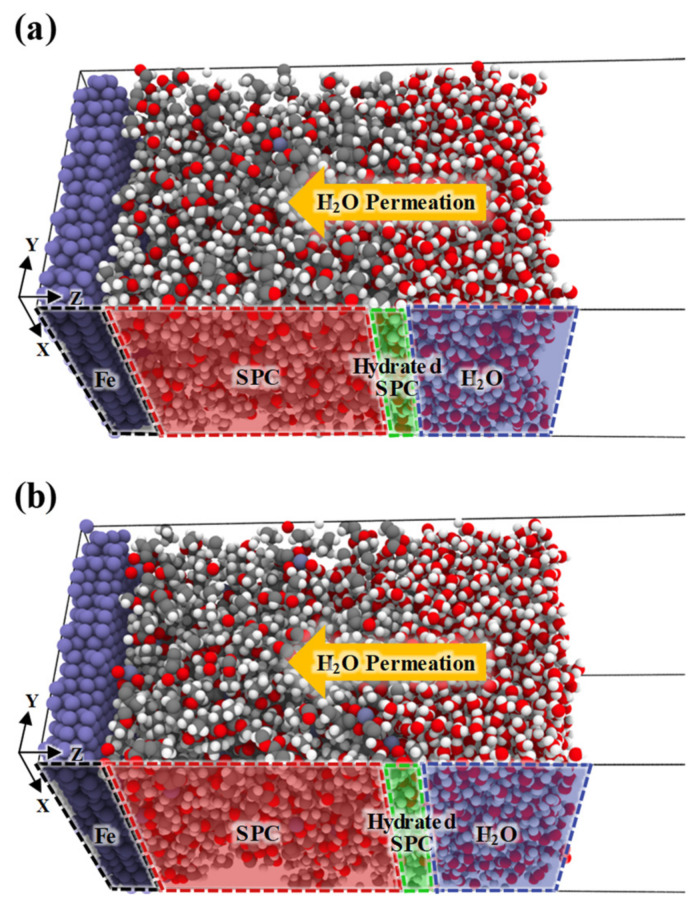
Initial equilibrated structures of (**a**) Z2 and (**b**) Z16 SPCs with H_2_O molecules on Fe slabs. White, gray, red, navy blue, and dark purple correspond to hydrogen, carbon, oxygen, zinc, and iron, respectively.

Figure 3 shows the initial structures of Z2 and Z16 with H_2_O molecules on the Fe(100) surface. The SPC and H_2_O regions are distinguished by SPC and H_2_O bulk densities greater than 90% [42,43]. The H_2_O molecules located over the SPC interface gradually permeate into the SPC due to the hydrolysable pendant groups, such as the ZMA moieties in the SPC, that attract H_2_O molecules. In particular, ZMA is strongly attracted to H_2_O through strong intermolecular interactions [16].

**Figure 4:**
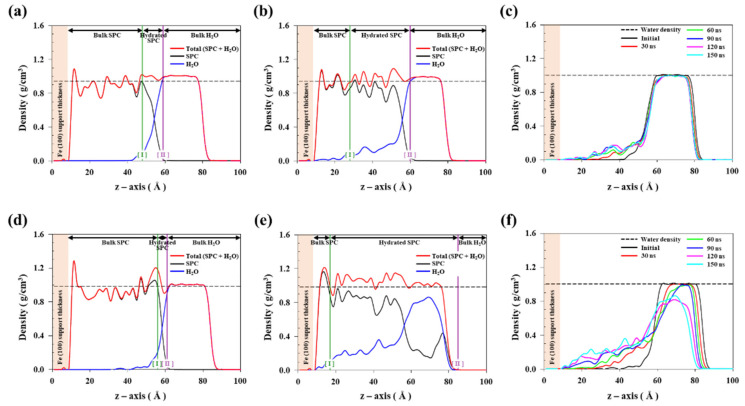
Density distributions of the sums of hydrated SPC + H_2_O molecules (red), SPC (black), and H_2_O molecules (blue): initial (**a**) Z2 and (**d**) Z16 states, and final (**b**) Z2 and (**e**) Z16 states. The black dash line shows the density of bulk SPC. The highlighted [I] and [II] lines delineate the bulk SPC and H_2_O regions, respectively, based on 90% of the bulk states of SPC and H_2_O molecules. Density distributions of H_2_O molecules in (**c**) Z2 and (**f**) Z16 over time (initial to 150 ns). The black dashed line shows the density of bulk H_2_O molecules.

To quantitatively analyse H_2_O permeation into the SPC on the Fe(100) surface, we analysed the time-evolution of the density profiles of Z2 and Z16. Figure 4 shows the initial and final SPC and H_2_O density distributions on Fe(100) surfaces. Figure 4a,d reveal that H_2_O is initially mainly located on the SPC surface, while Figure 4b,e show that the hydrated SPC regions gradually become spread as H_2_O molecules gradually permeate into the SPC. There are more H_2_O molecules in the hydrated Z16 SPC than that in Z2 because the higher proportion of ZMA in Z16 contributes to stronger interactions with adsorbed H_2_O molecules. Figure 4c,f show how the density distributions of the H_2_O molecules in Z2 and Z16 evolve in the 0–150 ns time period; H_2_O molecules located on the SPC surface gradually permeate into the SPC. In particular, the H_2_O molecules in Z16 permeate faster into the SPC than in Z2; hence, H_2_O is more soluble in Z16.

**Figure 5:**
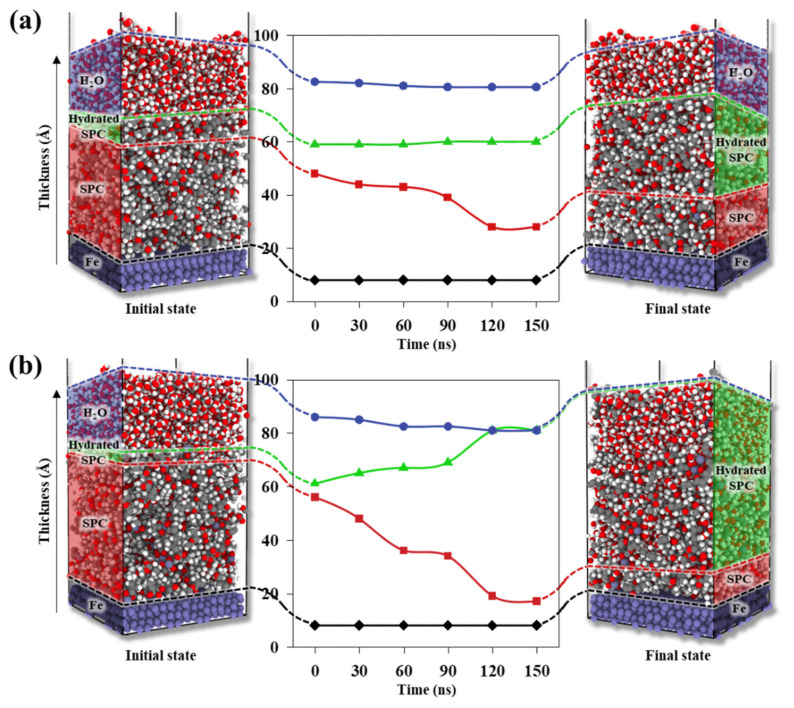
Snapshots of initial and final equilibrated structures of (**a**) Z2 and (**b**) Z16 with H_2_O molecules on Fe slabs. The regions (Fe, SPC, hydrated SPC, and H_2_O) are distinguished by the permeation of H_2_O molecules into SPC over time. White, gray, red, navy blue, and dark purple correspond to hydrogen, carbon, oxygen, zinc, and iron, respectively.

Figure 5 shows snapshots of initial and final equilibrated structures of Z2 and Z16 with H_2_O molecules on Fe(100) surfaces, which reveal that H_2_O permeation characteristics evolve over time. The hydrated SPC region gradually expands through the permeation of H_2_O molecules into the SPC; at the same time, the SPC polymer region gradually shrinks. Figure 5a shows that the hydrated SPC region expands toward the SPC region because the H_2_O permeates more slowly into Z2 than Z16. Consequently, the difference in the thicknesses of the H_2_O regions of the initial and final states of the Z2 model is only 5 Å, which indicates that H_2_O molecules are barely adsorbed into the SPC region. The finally hydrated SPC is 31 Å thick. Figure 5b shows that H_2_O molecules permeate into the SPC region of Z16 faster than in Z2. Moreover, the entire H_2_O region is adsorbed into the SPC, resulting in a final hydrated SPC that is 65 Å thick, ~2.1-times thicker than that of Z2. This swelling behaviour reveals that while the high content of ZMA in Z16 results in faster H_2_O adsorption, SPC polymers leach into the H_2_O region due to strong interactions between H_2_O and ZMA molecules. This heavier H_2_O permeation indicates that the swelling and permeation characteristics of SPC depend on the proportion of hydrolysable pendant groups in the SPC material.

### 3.3. H_2_O Permeability into SPC

The H_2_O and SPC density profiles accurately describe the characteristics of the SPC, such as the changing adsorption behaviour of the H_2_O molecules. In particular, H_2_O permeability is important because SPC is composed of hydrolysable polymers. The biocidal activity requires the hydrolysis and decomposition of the pendant groups, which, in turn, requires H_2_O permeation. Therefore, since the ZMA content changes with time, the permeability coefficient was calculated using Equation (2).

**Figure 6:**
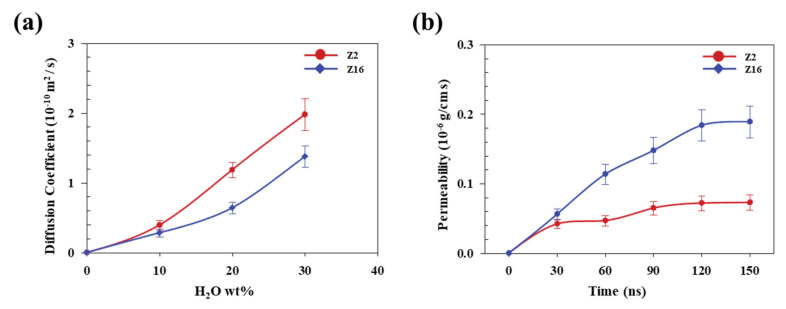
(**a**) H_2_O Diffusion coefficients of Z2 and Z16, and (**b**) H_2_O permeabilities of Z2 and Z16 over time.

(2) P=D×S

where *P*, *D*, and *S* are the permeability coefficient of H_2_O, the diffusion coefficient of H_2_O, and the solubility of H_2_O in the SPC system, respectively. The diffusion coefficient of H_2_O in the SPC was calculated using bulk-state SPC models. Figure 6a shows the H_2_O diffusion coefficients of Z2 and Z16 in SPCs with H_2_O molecules. The self-diffusion coefficient of H_2_O in the SPC model was calculated using Equation (3).

(3) $$D=\underset{t\to \infty }{\lim}\frac{1}{6t}{\left(r\left(t\right)-r\left(0\right)\right)}^{2}$$

where $r\left(t\right)$ and $r\left(0\right)$ represent the positions of H_2_O molecules at time t and t = 0, respectively. The diffusion coefficient of H_2_O was found to increase with increasing H_2_O content; they were (0.394 ± 0.069) × 10^−10^ cm^2^/s, (1.185 ± 0.107) × 10^−10^ cm^2^/s, and (1.979 ± 0.231) × 10^−10^ cm^2^/s for Z2 with H_2_O contents of 10, 20, and 30 wt%, respectively. In addition, the analogous values for Z16 were determined to be (0.282 ± 0.058) × 10^−10^ cm^2^/s, (0.639 ± 0.084) × 10^−10^ cm^2^/s, and (1.377 ± 0.156) × 10^−10^ cm^2^/s for H_2_O contents of 10, 20, and 30 wt%, respectively. Z2 exhibited higher diffusion coefficients than Z16 because the H_2_O molecules in Z16 are more constrained through strong interactions with ZMA molecules. Therefore, the H_2_O diffusion coefficient is highly dependent on the proportion of ZMA in the SPC.

Figure 6b shows relationships between the Z2 and Z16 permeabilities and time. H_2_O permeability gradually increased to 120 ns in both the Z2 and Z16 models and converged at 120–150 ns. The equilibrated permeability of Z16 was found to be ~2.5-times higher than that of Z2, which means that, despite the low diffusion coefficient of Z16, the permeability of Z16 is significantly higher than that of Z2 because H_2_O is very soluble in SPC, a result of strong ZMA/H_2_O interactions. Therefore, SPC permeability is strongly affected by ZMA content, with higher permeability achieved by increasing the ZMA content. A high permeability reveals that H_2_O molecules permeate faster into ZMA, with H_2_O molecules easily contacting the decomposition points near the metal pendant groups of the ZMA molecules. In addition, changes in the ZMA content affect biocidal performance through hydrolysis and decomposition, which affects antifouling behaviour in seawater.

## 4. Conclusions

The solubility and permeability of SPC were investigated as functions of ZMA content using MD simulations. SPC slab structures with H_2_O on Fe(100) surfaces were constructed to analyse the permeability of H_2_O into SPC by calculating the solubility and density distribution of H_2_O. Z16 was found to be more permeable than Z2 over time because, while H_2_O diffuses more into Z2 than Z16, H_2_O is more soluble in Z16 than Z2 due to strong H_2_O/ZMA interactions. In addition, the H_2_O permeation characteristics were found to depend on the ZMA content, with Z16 observed to swell more than Z2 due to the higher permeation of H_2_O into the SPC. This means that the H_2_O molecules in Z16 are more able to contact the decomposition point in the ZMA molecules than in Z2, which triggers biocidal activity through hydrolysis and decomposition in seawater. This study provides insights into how the performance of an SPC changes with ZMA content using MD simulations and provides an understanding of how antifouling agents protect vessels in seawater.

## Figures and Tables

**Table 1:** Proportions of various of antifouling agents in the SPCs in this study.

Agent	EA	MMA	2-MTA	n-BMA	ZMA
Z2 (mol%)	54	26	4	14	2
Z16 (mol%)	54	12	4	14	16

## Data Availability

Not applicable.
